# Phylogenomics revealed migration routes and adaptive radiation timing of Holarctic malaria mosquito species of the Maculipennis Group

**DOI:** 10.1186/s12915-023-01538-w

**Published:** 2023-04-10

**Authors:** Andrey A. Yurchenko, Anastasia N. Naumenko, Gleb N. Artemov, Dmitry A. Karagodin, James M. Hodge, Alena I. Velichevskaya, Alina A. Kokhanenko, Semen M. Bondarenko, Mohammad R. Abai, Maryam Kamali, Mikhail I. Gordeev, Anton V. Moskaev, Beniamino Caputo, Sargis A. Aghayan, Elina M. Baricheva, Vladimir N. Stegniy, Maria V. Sharakhova, Igor V. Sharakhov

**Affiliations:** 1 Department of Entomology, the Fralin Life Sciences Institute, Virginia Polytechnic Institute and State University, Blacksburg, VA USA; 2 Kurchatov Genomics Center, the Federal Research Center, Institute of Cytology and Genetics, Novosibirsk, Russia; 3 Current Address: INSERM U981, Gustave Roussy Institute, Université Paris-Saclay, Villejuif, France; 4 Department of Genetics and Cell Biology and the Laboratory of Ecology, Genetics and Environmental Protection, Tomsk State University, Tomsk, Russia; 5 Laboratory of Cell Differentiation Mechanisms, the Federal Research Center, Institute of Cytology and Genetics, Novosibirsk, Russia; 6 Department of Medical Entomology and Vector Control, Tehran University of Medical Sciences, Tehran, Iran; 7 Department of Medical Entomology and Parasitology, Tarbiat Modares University, Tehran, Iran; 8 Department of General Biology and Ecology, State University of Education, Mytishchi, Russia; 9 Dipartimento Di Sanità Pubblica E Malattie Infettive, Università Sapienza, Rome, Italy; 10 Scientific Center of Zoology and Hydroecology, National Academy of Sciences of the Republic of Armenia, Yerevan, Armenia; 11 Department of Zoology, Yerevan State University, Yerevan, Armenia

**Keywords:** Anopheles, Chromosomes, Introgression, Maculipennis Subgroup, Malaria vectors, Migration, Mosquitoes, Phylogenomics, Species radiation

## Abstract

**Background:**

Phylogenetic analyses of closely related species of mosquitoes are important for better understanding the evolution of traits contributing to transmission of vector-borne diseases. Six out of 41 dominant malaria vectors of the genus *Anopheles* in the world belong to the Maculipennis Group, which is subdivided into two Nearctic subgroups (Freeborni and Quadrimaculatus) and one Palearctic (Maculipennis) subgroup. Although previous studies considered the Nearctic subgroups as ancestral, details about their relationship with the Palearctic subgroup, and their migration times and routes from North America to Eurasia remain controversial. The Palearctic species *An. beklemishevi* is currently included in the Nearctic Quadrimaculatus subgroup adding to the uncertainties in mosquito systematics.

**Results:**

To reconstruct historic relationships in the Maculipennis Group, we conducted a phylogenomic analysis of 11 Palearctic and 2 Nearctic species based on sequences of 1271 orthologous genes. The analysis indicated that the Palearctic species *An. beklemishevi* clusters together with other Eurasian species and represents a basal lineage among them. Also, *An. beklemishevi* is related more closely to *An. freeborni*, which inhabits the Western United States, rather than to *An. quadrimaculatus*, a species from the Eastern United States. The time-calibrated tree suggests a migration of mosquitoes in the Maculipennis Group from North America to Eurasia about 20–25 million years ago through the Bering Land Bridge. A Hybridcheck analysis demonstrated highly significant signatures of introgression events between allopatric species *An. labranchiae* and *An. beklemishevi*. The analysis also identified ancestral introgression events between *An. sacharovi* and its Nearctic relative *An. freeborni* despite their current geographic isolation. The reconstructed phylogeny suggests that vector competence and the ability to enter complete diapause during winter evolved independently in different lineages of the Maculipennis Group.

**Conclusions:**

Our phylogenomic analyses reveal migration routes and adaptive radiation timing of Holarctic malaria vectors and strongly support the inclusion of *An. beklemishevi* into the Maculipennis Subgroup. Detailed knowledge of the evolutionary history of the Maculipennis Subgroup provides a framework for examining the genomic changes related to ecological adaptation and susceptibility to human pathogens. These genomic variations may inform researchers about similar changes in the future providing insights into the patterns of disease transmission in Eurasia.

**Supplementary Information:**

The online version contains supplementary material available at 10.1186/s12915-023-01538-w.

## Background

The genomics era offers new opportunities for a better understanding of the evolutionary history of species. Unlike traditional molecular phylogenetics, which relies on comparison of only a few sequenced markers, phylogenomics employs a large amount of genomic data based on thousands of single nucleotide polymorphisms (SNP) in the orthologous gene sequences. This feature makes phylogenomics a powerful and accurate tool for studying evolutionary relationships among groups of organisms involved in the transmission of human pathogens, such as mosquitoes [1–5]. Phylogenies can affect the interpretation of results from population genomics studies such as shared genetic variation and the detection of signatures of selection. For example, variations shared with basal lineages of phylogenetic trees would be interpreted as ancestral. Phylogenomics helps scientists better understand the evolution of epidemiologically important traits including host-seeking behavior, competence to pathogens, and adaptation of malaria mosquitoes to the natural environment [6–8]. The discovery of extensive introgression between species in the *Anopheles gambiae* complex [7] and the *An. funestus* complex [9] cautions against relying exclusively on a few genetic markers (such as single nuclear genes or mtDNA) for interpreting interspecific relationships in closely related anopheline mosquitoes.

The Maculipennis Group of malaria mosquitoes has the Holarctic distribution, which covers North America, Europe, Asia, and North Africa [10, 11]. According to the modern classification, the Maculipennis Group is subdivided into two Nearctic subgroups, the Freeborni and Quadrimaculatus Subgroup, and one Palearctic Maculipennis Subgroup [10, 12]. The group belongs to the subgenus Anopheles, which is the only cosmopolitan subgenus among anophelines [13]. The Maculipennis Group consists of 11 currently recognized Palearctic species: *Anopheles artemievi* Gordeyev, Zvantsov, Goryacheva, Shaikevich & Yezhov, 2005; *An. atroparvus* Van Thiel, 1972; *An. beklemishevi* Stegniy & Kabanova, 1976; *An. daciae* Linton, Nicolescu & Harbach, 2004; *An. labranchiae* Falleroni 1926; *An. maculipennis* Meigen, 1818; *An. martinius* Shingarev, 1926; *An. melanoon* Hackett, 1934; *An. messeae* Falleroni, 1926; *An. persiensis* Linton, Sedaghat & Harbach, 2003; and *An. sacharovi* Favre, 1903. Six out of 41 dominant malaria vectors in the world belong to the Maculipennis Group: *An. atroparvus*, *An. labranchiae*, *An. messeae*, and *An. sacharovi* in Eurasia, as well as *An. freeborni* Aitken, 1939 and *An. quadrimaculatus* Say, 1824 in North America [14, 15]. These six species are highly susceptible to various strains of the malaria parasite *Plasmodium vivax* [16–19]. Moreover, *An. messeae*, *An. daciae*, and *An. beklemishevi* were identified as the vectors of *Dirofilaria* parasitic nematodes that infect dogs and humans [20–23]. In addition, *An. atroparvus* has been implicated in transmission of the myxomatosis virus to domestic rabbits in the UK [24, 25]. Systematics studies of the Maculipennis Group, the former *Anopheles maculipennis* complex, have a long history. Originally, the phenomenon of “anophelism without malaria” in Europe led to the conclusion that *An. maculipennis* represents a complex of species with different abilities to transmit malaria [26, 27]. Later, J. Kitzmiller introduced the idea that the origin of the Maculipennis complex in the American tropics was followed by a migration of these mosquitoes from America to Eurasia with further radiation, producing the Palearctic group of species [28]. This idea was based on results from interspecies hybridization within and between the Palearctic and Nearctic members of the group. Studies of reproductive isolation demonstrated that hybridization between Palearctic members produced more viable and fertile progeny than hybridization between Nearctic members, suggesting that the Palearctic species radiated more recently [28–30]. The presence of fertile F1 females in some inter-species crosses points to the possibility of genetic introgression among the members of the Maculipennis Group. However, details about the phylogenetic relationships between and within these subgroups, a possibility of inter-species gene flow, and the routes and times of their migration from North America to Eurasia remain uncertain.

**Fig. 1:**
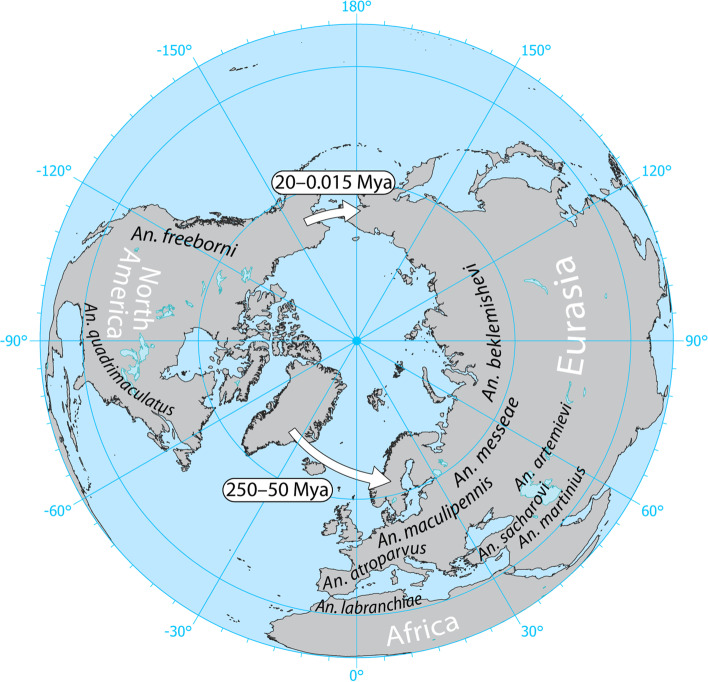
The hypothesis of two migration events of the Maculipennis from North America to Eurasia. The hypothesis was based on phylogenetic relationships between the Maculipennis species revealed by a cytogenetic analysis of their polytene chromosomes. Arrows show possible paths of migration that may have occurred in different times when the two continents were connected through Greenland about 250–50 Mya or through the Bering Land Bridge about 20–0.015 Mya

The taxonomy of the Maculipennis Group based on morphology is very difficult as its members have identical larval and adult characteristics and some of them can be identified only by the differences in the structure of the eggs [31]. The use of cytogenetics methods significantly contributed to species identification. Analyses of polytene and mitotic chromosomes described species-specific features of the mosquito karyotypes such as fixed inversions and heterochromatin structure [28, 32–36]. Cytogenetic photomaps for Palearctic species (*An. atroparvus*, *An. beklemishevi*,* An. labranchiae*,* An. maculipennis*, *An. martinius*,* An. melanoon*,* An. messeae*, and *An. sacharovi*) have been used to identify fixed overlapping chromosomal inversions [32]. These inversions were employed for the reconstruction of phylogenetic relationships among the members of the Maculipennis Subgroup [37]. Based on the assumption of the monophyletic origin of inversions, the Eurasian mosquitoes were divided into three major clades: the European clade (*An. labranchiae–An. atroparvus*), the Northern Eurasian clade (*An. melanoon–An. maculipennis–An. messeae*), and the Southern Eurasian clade (*An. sacharovi–An. martinius*) [32]*.* Following the principles of phylogeny reconstruction developed by Hennig [38], V.N. Stegniy suggested that if several species are identical in the structure of any chromosomal rearrangements, then that structure is phylogenetically ancestral in comparison with any other unique inversion form. Accordingly, the European clade was defined as the basal clade that gave rise to the Northern Eurasian and Southern Eurasian clades [32, 39]. The Eurasian species *An. beklemishevi* was not included in any of these clades since its chromosomal banding pattern was quite distinct. Although a cytogenetic analysis failed to establish phylogenetic relationships between *An. beklemishevi* and the other Palearctic members, similarities in the banding patterns were noticed between *An. beklemishevi* [32, 33] and the Nearctic species *An. earlei* Vargas, 1943 [40] from the Freeborni subgroup. The cytogenetic observations, the geographic distribution of the clades [32, 39], and the results of inter-species hybridization [28–30] led V.N. Stegniy to the hypothesis that two independent radiation events of malaria mosquitoes occurred from North America to Eurasia [37] (Fig. 1)*.* According to this hypothesis, the first migration of an ancestral species related to the eastern North American species *An. quadrimaculatus* occurred through the Greenland connection between Europe and eastern North America, which existed until the end of the Eocene, ~ 50 million years ago (Mya) [41]. This migration resulted in the origin of the European clade that further radiated into the Northern Eurasian and the Southern Eurasian clades. The second migration of an ancestral species related to *An. freeborni* occurred through the Bering Land Bridge, which existed between Asia and western North America ~ 20 Mya [41]. This migration gave rise to *An. beklemishevi.* An ecological study provided indirect support to the idea that *An. beklemishevi*, or its immediate ancestor, migrated into Eurasia via the Bering Land Bridge where the species’ range underwent substantial expansion [42].

Early molecular phylogenetic studies employed Internal Transcribe Spacer 2 (ITS2) sequences of ribosomal DNA to infer phylogenetic relationships between Palearctic and Nearctic members of the Maculipennis Group and provided support for the basal position of species from the Southern Eurasian clade rather than species from the European clade [43–45] as the cytogenetic studies suggested. Two of the molecular phylogenetic studies included ITS2 sequences from *An. beklemishevi* and pointed to the exceptional position of this species with respect to the Maculipennis Subgroup [44, 45] in agreement with the cytogenetic studies. R. Harbach, in 2004, proposed a classification for the *Anopheles* genus that was largely based on ITS2 sequence analyses [10]. According to this classification, the Maculipennis Group was subdivided into two Nearctic subgroups: Freeborni and Quadrimaculatus, and one Palearctic Maculipennis Subgroup. *Anopheles beklemishevi*, the species with the most northern distribution in Eurasia, was included in the Quadrimaculatus subgroup distributed in the southeastern areas of the North American biogeographical region. However, this placement contradicted the fact that the banding patterns of the polytene chromosomes are more similar between this species and *An. earlei* [37, 40], which belongs to the Freeborni subgroup [10]. More recent molecular studies of ITS2 sequences identified new Palearctic members of the Maculipennis Group including *An.* *artemievi* [46], *An. daciae* [47], and *An.* *persiensis* [48]. This expanded number of species added to the uncertainties in the phylogeny of the Maculipennis Subgroup.

In this study, we addressed several questions to clarify the historic relationships among the members of the Maculipennis Group by pursuing the following aims. (1) Reconstruct the phylogeny and identify the basal clade within the Maculipennis Subgroup. (2) Clarify the phylogenetic placement of *An. beklemishevi*. (3) Determine radiation times of the Palearctic and Nearctic members and suggest the likely scenario of species migration and radiation in the Maculipennis Subgroup. (4) Test for genetic introgression between the species. In order to achieve these aims, we used two independent approaches: a genome-wide molecular phylogeny and a rearrangement-based phylogeny centered on the gene orders on the X chromosome and fixed chromosomal inversions in the autosomes. Our study identified phylogenetic relationships within the Maculipennis Subgroup, clarifies the historic relationships between Nearctic and Palearctic members, determined the pattern of gene flow between the species, and helped to better understand the route and time of their migration between the continents. The reconstructed phylogeny suggests that vector competence and the ability to enter complete diapause during winter evolved independently in different lineages of the Maculipennis Group.

## Results

### * De novo genome and transcriptome assemblies of mosquitoes from the Maculipennis Group*

To generate the phylogeny of the Palearctic members of the Maculipennis Group, we sequenced genomes or transcriptomes of 10 Palearctic species and 2 Nearctic species. In addition, we used the available genome sequence of the Palearctic species *An. atroparvus* [49]. The genome sequencing reads for 4 species (*An. martinius*,* An. artemievi*,* An. melanoon*, and *An. persiensis*) were aligned to the *An. atroparvus* AatrE3 reference genome [49]. The mapping rate was high with 82.08%, 83.4%, 82.11%, and 84.89% of the paired reads properly aligned for *An. martinius*,* An. artemievi*,* An. melanoon*, and *An. persiensis*, respectively. We sequenced the transcriptomes of 8 species (*An. messeae*,* An. daciae*,* An. quadrimaculatus*,* An. beklemishevi*,* An. labranchiae*,* An. macullipennis*,* An. freeborni*, and *An. sacharovi*). Transcriptomes from the two geographically distant populations of *An. daciae* in Europe and Asia (Moscow and Tomsk regions, respectively) were also obtained and compared. After transcriptome assembly and annotation using the Transdecoder pipeline, we obtained between 14,404 and 22,326 proteins per species (Additional file 1: Table S1), with contig N50 ranging from 681 to 1329 bp and the total coding sequence (CDS) lengths ranging from 9.38 to 21.5 Mbp, which are typical transcriptome assembly metrics for non-model organisms. The single-copy orthologs benchmarking against the Diptera dataset demonstrated that 28.2–73.3% of genes per species were completely assembled during the analysis and 12.8–21.8% were fragmented. The duplication level, which can arise as a result of a separate assembly of different haplotypes especially in highly polymorphic species, was generally low and ranged from 0.2% (*An. beklemishevi*) to 3.6% (*An. quadrimaculatus*). Orthofinder analysis returned 1271 single-copy orthologs, which were present in all 13 species. These genes were aligned and concatenated, resulting in a 1,643,691 bp-long alignment. After the removal of gapped regions, the final alignment consisted of 898,101 bp. JModelTest2 results demonstrated that the best substitution model for our dataset was GTR + G + I, which was used as GTRGAMMAI during RaxML analysis.

### Multigene phylogeny of the Maculipennis Subgroup

**Fig. 2:**
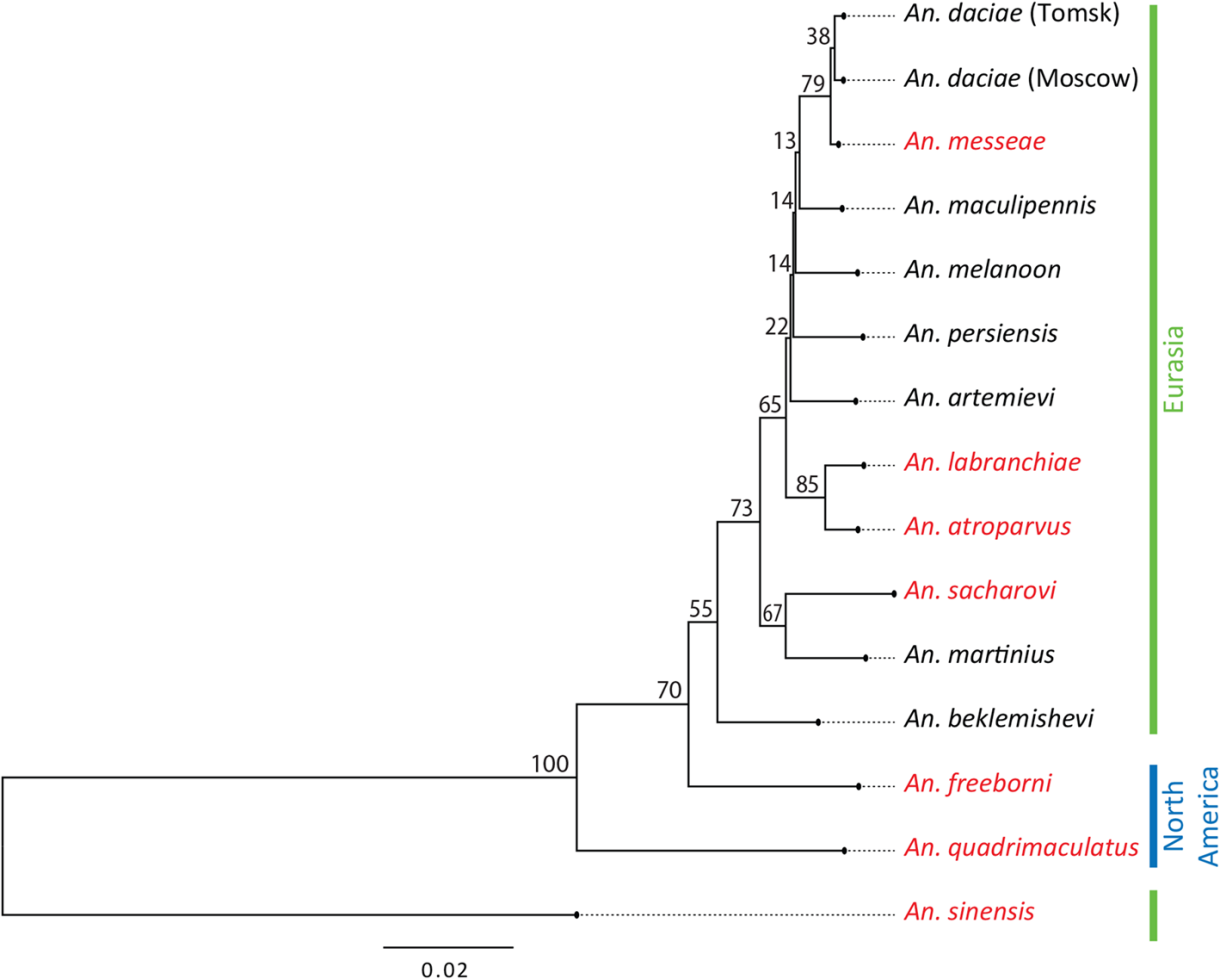
Maximum-likelihood tree of 14 *Anopheles* species based on the 1271 single-copy orthologs. Dominant malaria vectors are shown in red. Geographical distribution of the mosquitoes in Eurasia and North America is shown by lines with different colors on the right side of the figure. A scale bar refers to a phylogenetic distance in a fraction of nucleotide differences. All branches of the species tree have maximal bootstrap support (100%). The branch values indicate the percentage of individual orthogroup trees supporting the species tree

Maximum-likelihood tree reconstruction using RAxML demonstrated a high-confidence phylogeny with 100% bootstrap support for all the nodes (Fig. 2). The tree was rooted with *An. sinensis* Wiedemann, 1828 as an outgroup species. The analysis of the tree indicated that Palearctic and Nearctic members of the group represent separate phylogenetic branches. The placement of *An. beklemishevi* together with the Palearctic members of the group argues against the current systematic position of this species within the Nearctic Quadrimaculatus subgroup [10]. According to our phylogeny, *An. beklemishevi* belongs to the Maculipennis Subgroup. Moreover, *An. freeborni*, rather than *An. quadrimaculatus*, is more closely related to *An. beklemishevi* and other Eurasian species of the Maculipennis Subgroup. After migration of the Maculipennis mosquitoes to Eurasia and separation of the *An. beklemishevi* lineage, they further split into the Southern Eurasian clade (*An. sacharovi* and *An. martinius*), ancestors of the European clade (*An. atroparvus* and *An. labranchiae*) and the Northern Eurasian clade, which includes *An. artemievi*, *An. daciae*, *An. melanoon*, *An. persiensis*, *An. maculipennis*, and *An. messeae*. Interestingly, *An. daciae* from the Moscow region clusters together with *An. daciae* from the Tomsk region rather than with *An. messeae*, which was collected in the Moscow region. This result further supports the species status of *An. daciae* [2]. Additionally, we tested the phylogenetic concordance between individual gene trees. For the majority of nodes, the splits were supported by more than 50% of the orthogroups with the exception for intermediate nodes with recent divergence and/or influenced by introgression events or incomplete gene sorting. This is also evident from a separate analysis in which we split all ortholog groups into 4 equally-sized datasets based on the length of the alignments after *trimal* filtration. The analysis shows a clear trend of increasing of the phylogenetic concordance with longer alignments of orthogroups (Additional file 1: Fig. S1).

### Divergence times among species of the Maculipennis Subgroup

**Fig. 3:**
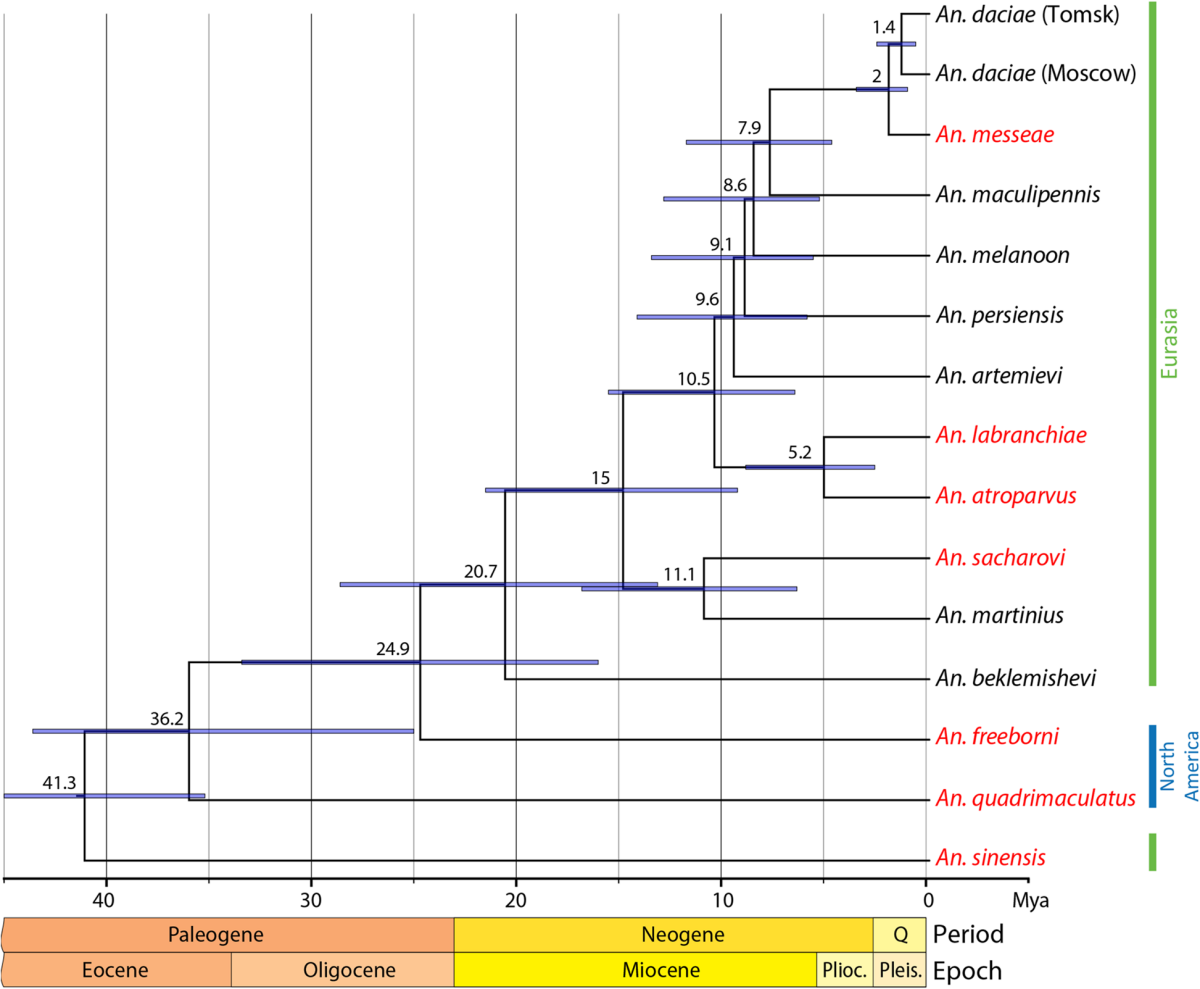
A time-calibrated phylogenetic tree for the studied species based on the 126,025 fourfold degenerated sites. The time scale is in Mya, mean values and time intervals are indicated in blue above the branches. Dominant malaria vectors are shown in red. Geographical distribution of the mosquitoes in Eurasia and North America is shown by lines with different colors on the right side of the figure. Q, Quaternary; Plioc., Pliocene; Pleis., Pleistocene

Our estimation of the divergence times demonstrated that the origin of the Maculipennis Group is dated back ~ 36.2 Mya when the Quadrimaculatus subgroup separated from the remaining species of the Maculipennis Group (Fig. 3). The split between the Nearctic and Palearctic species of the Maculipennis Group occurred between 16 and 34 Mya with a mean estimate of 24.9 Mya. Migration of mosquitoes from North America to Eurasia may have occurred before ~ 20.7 Mya in the Neogene Period (Miocene Epoch) followed by a split between the most northern species *An. beklemishevi* and the remaining species of the Maculipennis Subgroup. Around 15 Mya, in the mid-Miocene, a separation between the Southern Eurasian *An. sacharovi–An. martinius* clade and the remaining species occurred. Next, a split between the European clade and the Northern Eurasian clade took place around 10.5 Mya. The most recent species divergence occurred between *An. daciae* and *An. messeae* between 3.6 and 1.1 Mya (a mean estimate of 2 Mya) in the Pliocene–Pleistocene Epoch at the end of the Eogene Period. The mean divergence time of 1.4 Mya between the Tomsk and Moscow populations of *An. daciae* is probably an overestimation as population divergence time can be better assessed using population samples and coalescent analysis, taking into account migration events and demographic features of populations [50]. This analysis used previously established divergence time of the *An. sinensis* split from the rest of the species (35–45 Mya) [6]. To confirm the obtained tree topology and divergence times, we performed additional analysis using of the whole-genome dataset from only six species including the outgroup *An. sinensis* (Additional file 1: Fig. S2).

### Signatures of genomic introgression between species of the Maculipennis Group

**Fig. 4:**
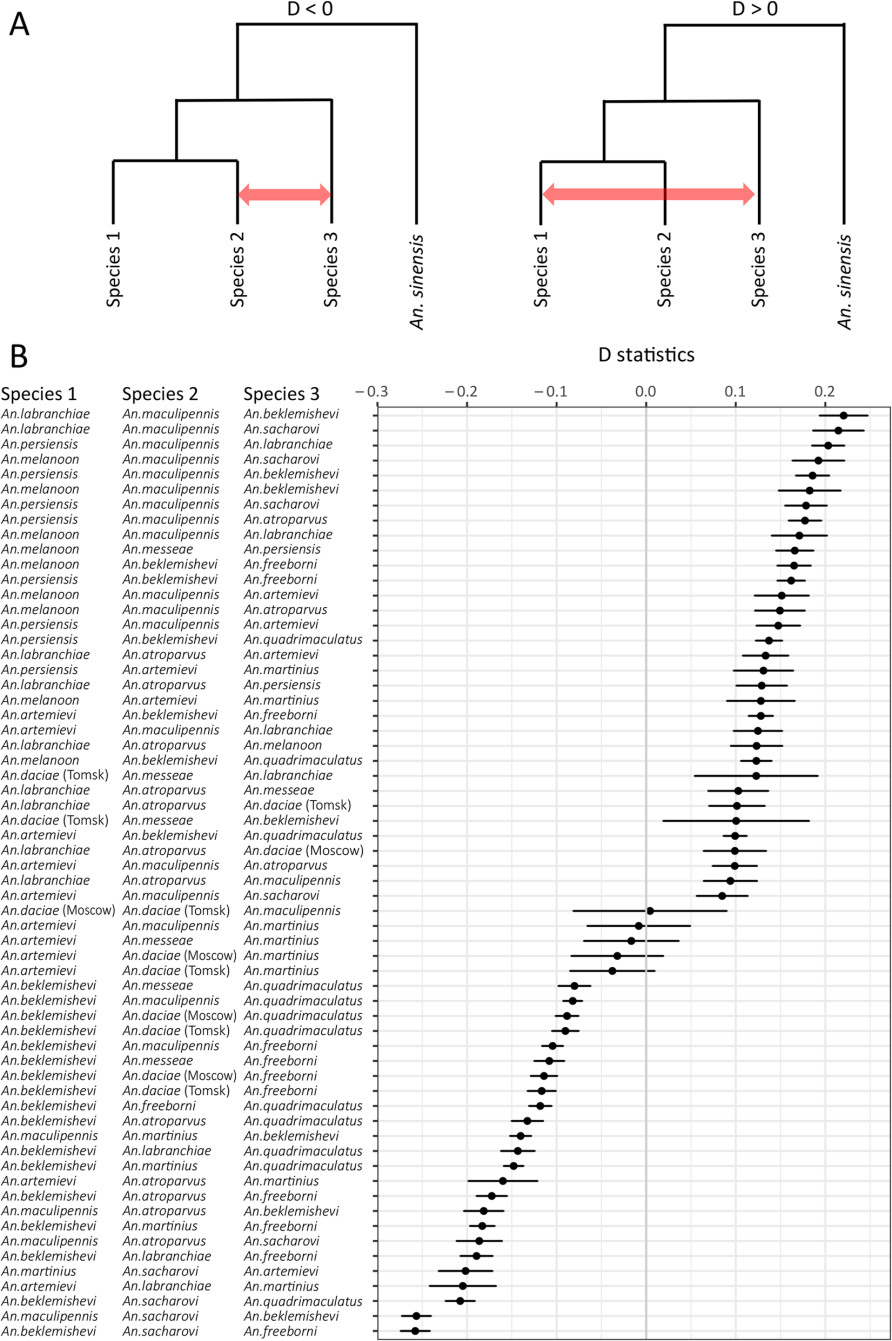
Signatures of genomic introgression among the species of the Maculipennis Group. **A** A schematic explanation of the analysis using D statistics. We tested for discordant genealogies given the maximum-likelihood species tree between species 2 and 3 and species 1 and 3. **B** Introgression statistics (D) among species shown on the left and their standard deviations (SD) shown by lines on the right. Only combinations of species with Bonferroni corrected *P*-values < 0.001 are shown. Negative values of D statistics imply gene flow between species 2 and 3, positive values between species 1 and 3, with *An. sinensis* as an outgroup as schematically depicted in panel **A**

To understand the pattern of a possible gene flow and introgression events, implying absence of complete reproductive isolation among members of the Maculipennis Group, we used the D statistics (ABBA-BABA test, 4-taxon) analysis (Fig. 4). Our study detected highly significant and widespread signatures of introgression events between the species within the group including species which are geographically isolated today such as *An. labranchiae* and *An. beklemishevi* or *An. freeborni* and *An. sacharovi*. The current phylogenetic setting of the analysis cannot unfortunately assess the level of introgression between closely related species such as *An. labranchiae* and *An. atroparvus* where population sampling is needed.

### Chromosome phylogeny of the Maculipennis Subgroup

To understand the karyotypic evolution within the Maculipennis Subgroup, we analyzed X chromosome rearrangements among five members and autosomal rearrangements among eight members. The banding patterns of X chromosomes could not be compared visually due to the high numbers of fixed rearrangements among the species [37, 39]. For this reason, the X chromosomal rearrangements were identified based on the order the genes mapped using fluorescence in situ hybridization (FISH). We selected 21 genes from the *An. atroparvus* genome [49] separated from each other by 208,734–1,320,538 bp (~ 800 kb on average) according to the genome map (Additional file 1: Table S2). The DNA probes were designed based on the exons of these genes.

**Fig. 5:**
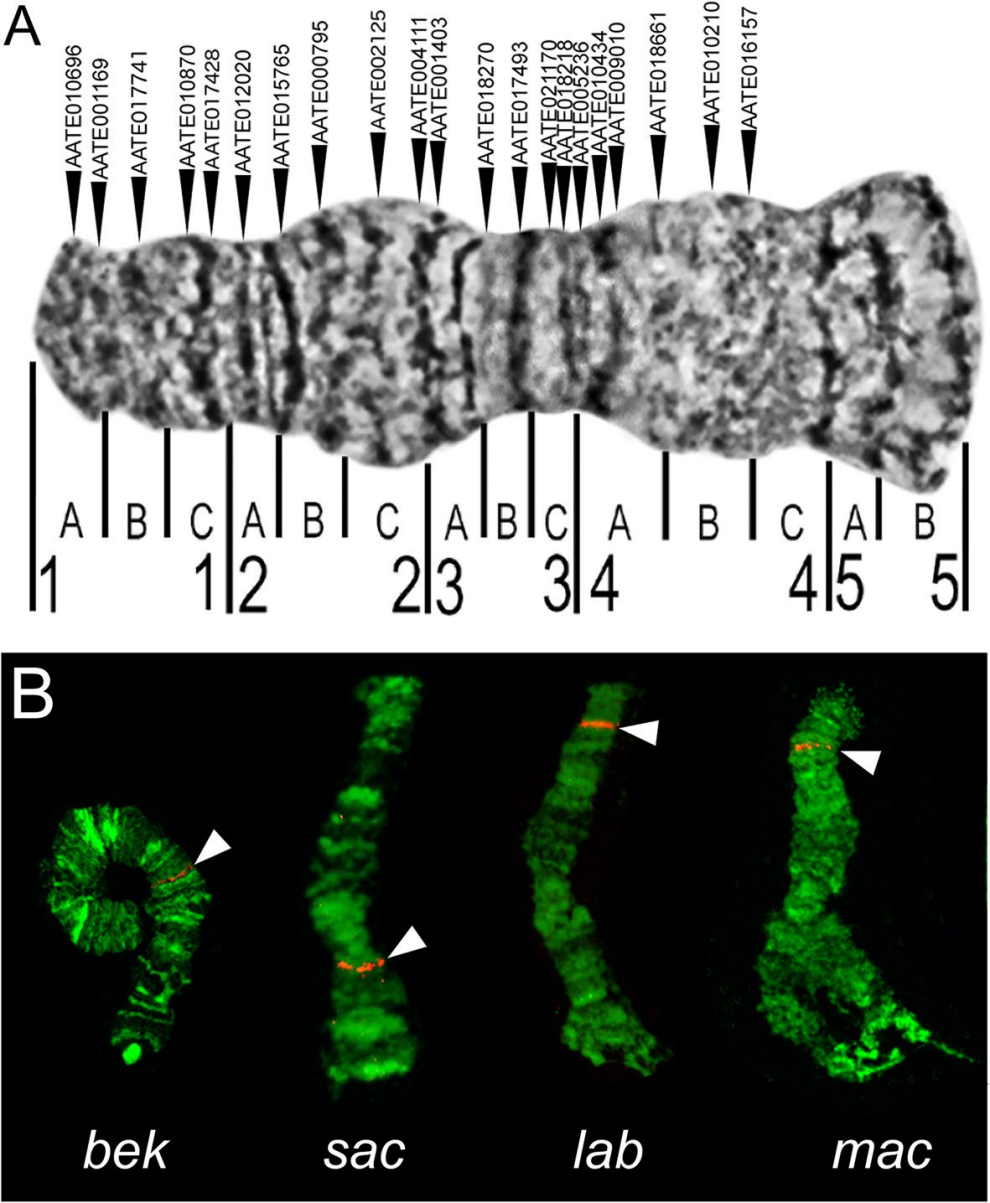
Physical mapping of marker genes on X chromosomes of species from the Maculipennis Group. Positions of 21 marker genes in the X chromosome of *An. atroparvus* (**A**) are shown by arrows above the chromosome. Numbers and letters below the chromosome indicate numbered divisions and lettered subdivisions. Localization of the gene AATE017741 in the X chromosome of *An. beklemishevi* (*bek*), *An. sacharovi* (*sac*), *An. labranchiae* (*lab*), and *An. maculipennis* (*mac*) are shown in panel **B**, indicating significant reshuffling of chromosome arrangements among the species

The probes were amplified, labeled, and hybridized to the polytene chromosome preparations from the ovarian nurse cells of *An. atroparvus* to confirm the order and distances between them on the X chromosome (Fig. 5A). Selected markers benchmarked ~ 75% of the physical length of the X chromosome from subdivisions 1A to 4B, representing the euchromatic part of the chromosome [51]. We hybridized the same probes with polytene chromosomes of *An. beklemishevi*,* An. labranchiae*, *An. maculipennis*, and *An. sacharovi*. Not all of the genes were successfully mapped to the chromosomes of all the species. We were able to hybridize and map all 21 genes only in *An. labranchiae* and *An. maculipennis*, 20 and 19 DNA probes hybridized to the chromosomes of *An. sacharovi* and *An. beklemishevi*, respectively. In total, 17 gene probes hybridized to all the analyzed species and were used for further analysis. Gene orders in three species, *An. labranchiae*, *An. atroparvus*, and *An. maculipennis*, were identical, while gene orders in the remaining species were different from each other. Differences among species in the chromosomal position of the gene marker AATE17741 are shown in Fig. 5B.

We calculated the number of X chromosome rearrangements and reconstructed phylogenetic relationships among seven species using the Multiple Genome Rearrangements (MGR) program [52]. Conserved gene orders were considered as synteny blocks. The pattern of synteny blocks in *An. atroparvus* was considered as the standard according to the previously established nomenclature [37]. The following orders and orientations of conserved genes and synteny blocks were used as an input for the MGR analysis.

 > *An. sacharovi* X.

1 -5 -4 3 -2 8 9 6 7

 > *An. atroparvus* X.

1 2 3 4 5 6 7 8 9

 > *An. labranchiae* X.

1 2 3 4 5 6 7 8 9

 > *An. maculipennis* X.

1 2 3 4 5 6 7 8 9

 > *An. beklemishevi* X.

1 3 4 8 -9 2 7 -6 -5

**Fig. 6:**
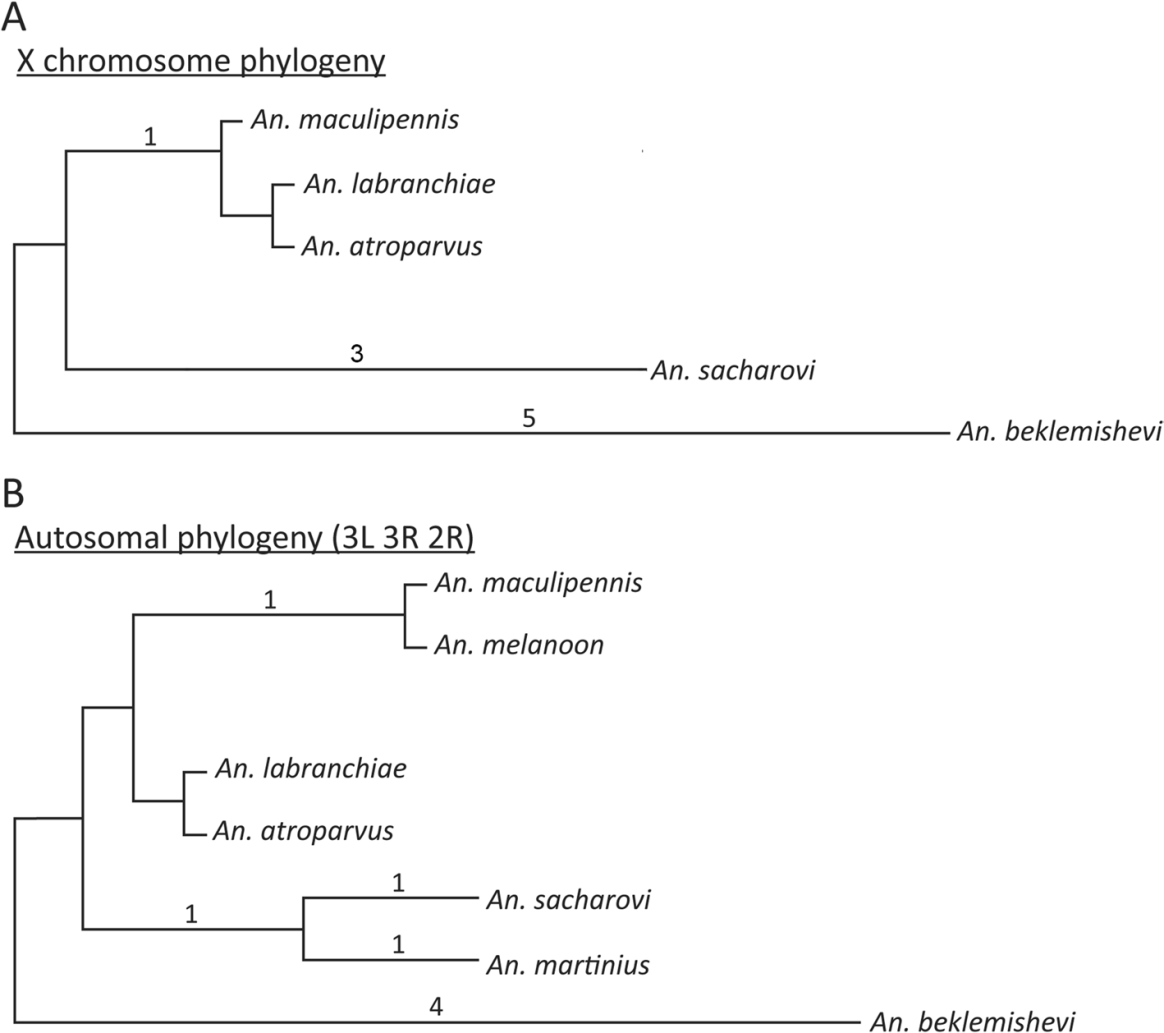
Chromosome-based phylogeny for the Maculipennis Group. **A** Rearrangement phylogeny based on positions of 17 DNA probes mapped to the X chromosomes of five species. **B** Rearrangement phylogeny based on autosomal banding pattern differences detected in eight species. Numbers above tree branches indicate the number of inversions fixed between the lineages

The MGR program reconstructed putative ancestral karyotypes and created a phylogenetic tree by implementing an algorithm that minimized the sum of the rearrangements over all edges of the phylogenetic tree (Fig. 6A). The program clustered together *An. atroparvus*, *An. labranchiae*, and *An. maculipennis* since they had no fixed inversions. *Anopheles sacharovi* was separated from *An. atroparvus* by four fixed inversions. Overall, the X chromosome rearrangement topology agrees with the whole-genome molecular phylogeny demonstrating that *An. beklemishevi* is the most distantly related species to the other members.

We also analyzed rearrangements on the autosomes in eight Palearctic members of the Maculipennis Subgroup. For this analysis, we used the chromosome banding patterns that were previously described by V.N. Stegniy [32, 37]. Chromosomes of *An. atroparvus*, *An. beklemishevi*,* An. labranchiae*, *An. maculipennis*, *An. martinius*, *An. melanoon*, *An. messeae*, and *An. sacharovi* were compared. Banding patterns in *An. atroparvus* were considered as the standard according to the previously established nomenclature [37]. Chromosomal rearrangements were determined in the 2R, 3R, and 3L autosomal arms. Banding patterns in two groups of species were identical: *An. atroparvus*–*An. labranchiae* and *An. maculipennis*–*An. melanoon–An. messeae*. The following orders and orientations of conserved synteny blocks on autosomes were used as an input for the MGR analysis.

 > *An. atroparvus* 3L 3R 2R.

1 2 3 4 5 6 7 8 9 10 11 12 13 14 15 16

17 18 19 20 21 22 23

24 25 26 27

 > *An. labranchiae* 3L 3R 2R.

1 2 3 4 5 6 7 8 9 10 11 12 13 14 15 16

17 18 19 20 21 22 23

24 25 26 27

 > *An. maculipennis* 3L 3R 2R.

1 2 3 4 5 6 7 8 9 10 11 12 13 14 15 16

17 -20 -19 -18 21 22 23

24 25 26 27

 > *An. melanoon* 3L 3R 2R.

1 2 3 4 5 6 7 8 9 10 11 12 13 14 15 16

17 -20 -19 -18 21 22 23

24 25 26 27

 > *An. messeae* 3L 3R 2R.

1 2 3 4 5 6 7 8 9 10 11 12 13 14 15 16

17 -20 -19 -18 21 22 23

24 25 26 27

 > *An. sacharovi* 3L 3R 2R.

1 2 3 -15 -14 -13 -12 -11 -10 -9 8 -7 -6 -5 -4 16

17 18 19 20 21 22 23

24 25 26 27

 > *An. martinius* 3L 3R 2R.

1 11 12 13 14 15 -3 -2 -10 -9 -8 -7 -6 -5 -4 16

17 18 19 20 21 22 23

24 25 26 27

 > *An. beklemishevi* 3L 3R 2R.

1 2 5 6 7 8 9 10 11 12 13 14 15 -4 -3 16

17 18 -22 -21 -20 -19 23

24 -26 -25 27

Overall, the topology of autosomal (Fig. 6B) and X chromosome phylogenetic trees (Fig. 6A) were very similar to each other. *Anopheles atroparvus* and *An. labranchiae* clustered together. The *An. maculipennis–An. melanoon–An. messeae* cluster was separated from the *An. atroparvus*–*An. labranchiae* cluster by one inversion. *Anopheles sacharovi* and *An. martinius* were separated from *An. atroparvus* by two inversions. In agreement with the X rearrangement and the molecular phylogenies, *An. beklemishevi* was the most distant from the remaining species.

## Discussion

### The revised phylogenetic placements of species in the Maculipennis Subgroup

In this study, we reconstructed a multigene phylogeny for the members of the Maculipennis Group. Our phylogeny, for the first time, included all 11 described Palearctic species together with two Nearctic species and demonstrated that the Palearctic members of the Maculipennis Group radiated more recently than the Nearctic members. Similar to the chromosomal [37, 39] and ITS2-based phylogenies [44], our analysis supported the presence of three major clades in the Maculipennis Subgroup: the Southern Eurasian clade of *An. sacharovi* and *An. martinius*, the European clade of *An. atroparvus* and *An. labranchiae*, and the Northern Eurasian clade of the remaining species. Similar to the previous chromosomal rearrangement phylogeny [37, 39] and the ITS2-based phylogenies [43–45], our phylogeny clusters together species with identical chromosomal patterns, *An. atroparvus* and *An. labranchiae* [28]. Unlike the ITS2-based phylogenies [43–45], our genome-wide phylogeny provides strong support for the branching order of all Eurasian species. The Southern Eurasian clade emerged as a basal phylogenetic branch with respect to the European clade and the Northern Eurasian clade.

The systematic position of *An. beklemishevi* remained controversial until our study. The current taxonomic nomenclature placed *An. beklemishevi*, a species with a broad geographic distribution in the most northern areas of Eurasia, into the Nearctic Quadrimaculatus subgroup [10]. This subgroup includes the dominant malaria vector *An. quadrimaculatus* [15] and four other species. *Anopheles quadrimaculatus* has a broad distribution in the eastern areas of the North American biogeographical region. The Nearctic Freeborni Subgroup includes *An. freeborni*, a dominant vector of malaria in the western areas of the North American biogeographical region [15], and three other species: *An. occidentalis*,* An. hermsi*, and *An. earlei* [10]. The geographic distribution of *An. freeborni*, *An. occidentalis*, and *An. hermsi* is restricted to the west coast of the North America. In contrast, *An. earlei* has a broad distribution in the northern areas in the North American biogeographical region. The cytogenetic analysis determined substantial differences in chromosomal banding patterns caused by the overlapping chromosomal inversions among the Palearctic members of the Maculipennis Group [28–30]. The phylogenetic position of *An. beklemishevi* remained unresolved because of the absence of an intermediate chromosome arrangement that could connect *An. beklemishevi* with other species. Nevertheless, comparison of its chromosomes with Nearctic members of the group suggested that *An. beklemishevi* is a close relative to *An. earlei* [40]. Our genome-based phylogeny strongly suggests that *An. beklemishevi* belongs to the most basal branch among the Palearctic members in the Maculipennis Group. Moreover, the Eurasian species are more closely related to *An. freeborni* than to *An. quadrimaculatus*. Therefore, our study strongly argues against placing *An. beklemishevi* into the Quadrimaculatus subgroup. Also, the autosomal and X chromosomal rearrangement phylogenies were similar to the multigene phylogeny and supported the most distant position of *An. beklemishevi* with respect to the other members of the Maculipennis Subgroup. Phylogenomic and cytogenetic analyses of the remaining Nearctic species will help to better resolve the systematic positions of all members of the Maculipennis Group.

Our phylogenetic tree allowed for the analysis of introgression events between the species. The introgression events were detected between many members of the group. For example, *An. beklemishevi* demonstrated significant signatures of introgression with allopatric species *An. labranchiae.* Also, the Nearctic species *An. freeborni* showed significant signatures of introgression with the Palearctic species *An. sacharovi*. The evidence of gene flow between allopatric species can be interpreted as historical events including those that occurred during the last Glacial Maximum in refugia regions when the species boundaries were shifted and differed significantly from the current distribution. Another explanation can be the current gene flow events across the species boundaries which permit genetic exchange between the species and their populations. In this situation, two allopatric species can demonstrate genetic admixture indirectly mediated by a third species which is sympatric for both of them. To prove the existence of such a scheme one will need to sample major populations from diverse geographic regions of several species. The phenomena of widespread historical introgression and current incomplete reproductive isolation have been demonstrated in the African *An. gambiae* complex using whole genome datasets [7]. It is possible that genetic introgression can be a factor contributing to the acquisition of adaptations related to malaria vectorial capacity. For example, a study of introgression between members of the *An. gambiae* complex suggested that traits enhancing vectorial capacity can be acquired from nonsister vector species through a rapid process of interspecific genetic exchange [7]. Similarly, a genomic analysis of the *An. funestus* complex demonstrated substantial gene flow among vector and nonvector species further supporting introgression as a common mechanism that facilitates adaptation to new environments and enhancing vectorial capacity in malaria mosquitoes [9].

The new phylogeny for the Maculipennis Subgroup provides a foundation for hypothesis generation and testing to further our understanding of evolution of the diverse biological traits that determine vectorial capacity. Among 13 studied members of the Maculipennis Group, six species — *An. messeae*, *An. labranchiae*, *An. atroparvus*, *An. sacharovi*,* An. freeborni*, and *An. quadrimaculatus* — are dominant vectors of malaria [14, 15]. However, the malaria vectors and nonvectors do not form two separate monophyletic groups. Thus, our phylogenomic analysis suggests that traits important for vectorial capacity (including vector competence and ecological adaptations) evolved multiple times in the evolution of the Maculipennis Group and likely depended on species distribution rather than on phylogenetic relationships. The independent origin of vector competence and ecological adaptations raises important questions. First, is the evolution of independently originated traits determined by changes of the same or different genomic loci in vector species? Second, what are specific nucleotide and amino acid substitutions that increase epidemiologically important traits in different mosquito species? Knowledge about genomic changes related to ecological and physiological plasticity and to susceptibility to pathogens in the Maculipennis Subgroup may inform us about the likelihood that similar changes will occur in the future.

### A working hypothesis of species radiation, migration, and adaptation in the Maculipennis Subgroup

Based on the current distribution of species from the Maculipennis Group and the phylogenies developed in this study, we propose that migration of the Maculipennis mosquitoes occurred through the Bering Land Bridge ~ 20.7 Mya during the Early Miocene. Because of the fluctuation of the sea level, this bridge existed intermittently across the Bering Straits, connecting Chukotka with Alaska and allowing animals to migrate between North America and Eurasia [41]. The connection between the two continents occurred from the Paleocene, ~ 60 Mya, through the Eocene, ~ 40 Mya, and from the Miocene, ~ 20 Mya, until relatively recent times. Various species of animals [53] including horses [54] and most recently ancient humans [55, 56] were able to use the Bering Land Bridge for their migration between the continents. The possible alternative scenario of the Maculipennis Group migration through Greenland is unlikely. It was originally proposed to explain the distribution of the majority of the species in Europe and Western Asia rather than in Eastern Asia and the Far East. Although the connection between North America and Eurasia through Greenland existed in the Paleocene ~ 60 Mya, it was disrupted in the Eocene [41] before mosquitoes migrated according to our phylogeny. Moreover, the basal species among the Palearctic mosquitoes, *An. beklemishevi*, is more closely related to *An. freeborni*, the species from the western area of the North American biogeographical region located close to the Beringia, rather than to *An. quadrimaculatus* from the eastern area of the North American biogeographical region.

**Fig. 7:**
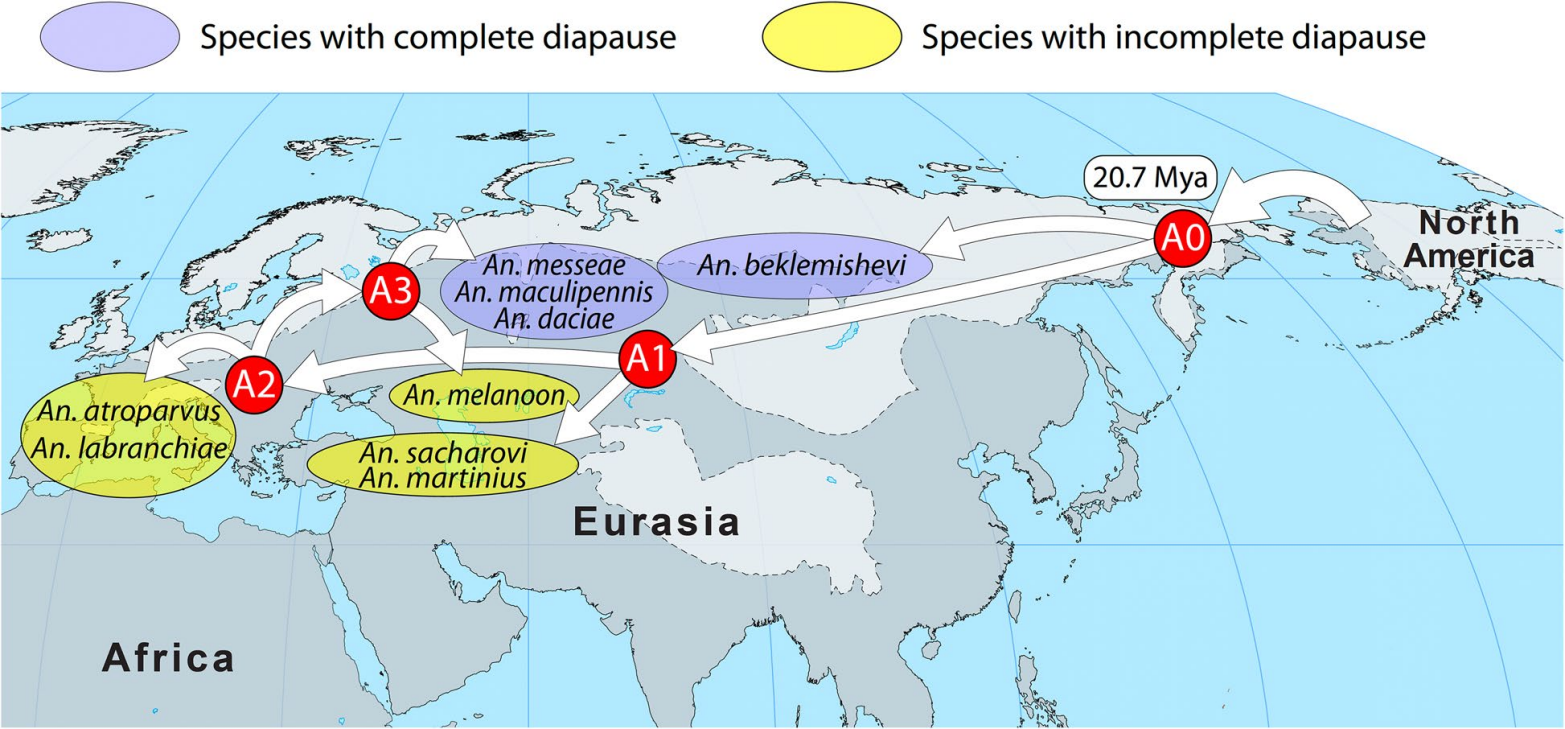
A scenario of the Maculipennis Group migration through the Bering Land Bridge. This scenario is supported by the existence of the land connection between Alaska and Chukotka at the time of mosquito migration and the split between *An. beklemishevi* and the rest of the Maculipennis Subgroup around 20.7 Mya. The last large glaciation (shown in light gray color) explains why the Maculipennis mosquitoes are not present in the Far East. Letters A0, A1, A2, and A3 in red circles represent hypothetical ancestral species for the phylogenetic clades. Species entering complete diapause with large lipid reserves are shown in blue ovals. The scheme does not reflect the actual extent of species distribution

Louis Agassiz’s work in the ninetieth century highlighted the importance of glaciation to biogeography of species [12]. With respect to the past glaciation events in the Northern Hemisphere, the Maculipennis mosquitoes can be divided into three groups: (1) species restricted to the northern parts, probably originating from glacial relict populations in gaps between ice sheets or colonized land freed from receding glaciers (e.g., *An. earlei* and *An. beklemishevi*); (2) species present both in the northern and southern regions, possibly representing mosquitoes that have been able to colonize the northern areas from the southern areas (e.g., *An. freeborni*,* An. quadrimaculatus*,* An. maculipennis*,* An. messeae*, and *An. daciae*); and (3) species restricted to the southern parts (e.g., *An. sacharovi*, *An. martinius*,* An. atroparvus*,* An. labranchiae*,* An. artemievi*, *An. persiensis*, and *An. melanoon*). Evolution of the Maculipennis Subgroup could have been affected by multiple glaciation events that occurred in Eurasia in the Neogene Period after 23 Mya [41]. We speculate that glaciation shaped the divergence and geographic distribution in the Palearctic Maculipennis species by pushing their natural ranges westward (Fig. 7). At the last glaciation maximum, the ice sheet covered northern Europe, Scandinavia, northeastern Asia, and a large part of North America [57]. Moreover, much of Siberia, the Far East, and Central Asia were desert-like with only ~ 2% of the ground covered by vegetation. The ice sheet distribution during the last glaciation period may explain the current absence of the Maculipennis mosquitoes from the eastern territories of Eurasia. Among the extant species of the Maculipennis Subgroup, adult females of *An. beklemishevi*,* An. maculipennis*,* An. messeae*, and *An. daciae* form large lipid reserves before entering complete diapause in winter, while *An. atroparvus*,* An. labranchiae*,* An. melanoon*, *An. martinius*, and *An. sacharovi* females do not develop large fat bodies but continue taking occasional blood meals during incomplete diapause in winter [42, 58–64]. Females that go into complete diapause are not likely to carry the *Plasmodium* infection over into the following spring, but females that go into incomplete diapause can continue transmitting malaria until late autumn [64]. We hypothesize that the ancestral species of the Maculipennis Subgroup arrived in Eurasia with a well-developed ability to enter complete diapause during winter. This species may have given rise to *An. beklemishevi*, which remained in the northern territories but had to move westward (Fig. 7). The origin of the Southern Eurasian clade, *An. sacharovi*–*An. martinius*, occurred when the overall temperature increased in the middle of the Miocene, 15 Mya [41] and these species developed incomplete diapause and the adaptation to survive in hot and arid climates. Other species reached Europe ~ 10.5 Mya and gave rise to the European clade and the Northern Eurasian clade. Among the species of the Northern Eurasian clade, *An. artemievi*, *An. persiensis*, and *An. melanoon*, adapted to the warmer climate. Around 8 Mya, when the planet’s temperature was cooling again, *An. maculipennis*,* An. messeae*, and *An. daciae* of the Northern Eurasian clade developed complete diapause and became sympatric with *An. beklemishevi*. All the species from this clade gradually moved toward the East. Interestingly, in species with a large distribution in Eurasia, such as *An. maculipennis*, northern populations can enter complete diapause to overwinter but southern *An. maculipennis* populations, like the southern species *An. sacharovi*, preserved their ability to bloodfeed during winter [61]. The most recent split between *An. messeae* and *An. daciae* overlapped with the strong glaciation period that occurred ~ 3 Mya [41]. Because of currently rising temperatures, the southern species of the Maculipennis Subgroup have been spreading northwards. For example, *An. maculipennis* recently extended its range to the Northeast and has reached the Southern Urals [65]. Overall, these observations suggest, that the ability to enter complete diapause in winter evolved independently in different lineages of the Maculipennis Group depending on the climate conditions. Similarly, chromosomal inversion-based [66–68] and multigene [6–8] phylogenies of the *An. gambiae* complex support independent evolution of physiological adaptation to breeding in saltwater in *An. merus* Dönitz, 1902 and *An. melas* Theobald, 1903.

Our study suggests that adaptation of malaria mosquitoes to different climates was an important factor in their evolution. The split of the Maculipennis Group into the Palearctic and Nearctic lineages coincided with the highest rate of speciation among Anophelinae mosquitoes in the world, which occurred around 30–24 Mya [3, 69]. This rapid speciation in mosquitoes correlates with the overall increases in concentration of atmospheric CO_2_, overall temperature on the planet, and diversification of mammals [69]. The elevated rate of mosquito speciation also overlapped with the evolution of grasses [70] that provided new habitats for the larval stages of mosquitoes and facilitated the diversification of mammal species, such as ruminants [71] and rodents [72], increasing the host availability for adult mosquitoes [69]. Given the importance of environmental factors for the distribution of mosquito species, the ongoing climate change may lead to the geographical redistribution of malaria vectors and return of malaria transmission to the territories where it was eliminated [73–77].

The phylogenomic analysis of the Maculipennis Group can inform similar analyses in other insect species. Northern taxa of many dipterans tend to be distributed widely across the Nearctic and Palearctic areas [78]. For example, a northern trans-Atlantic track has been described for the Keroplatidae fungus gnats [79] and the Chironomidae nonbiting midges [80]. Roles of both Beringia and the Mediterranean have been identified in generating geographic vicariant patterns for currently boreal insects [78]. Beringia has been implicated in linking eastern Asian with northwestern Nearctic carabid beetles. It has been proposed that all early interactions between the Palearctic and Nearctic species of the tipulid *Nephrotoma dorsalis* group involved Beringia, with two main vicariances dated to Oligocene and late Pliocene [81]. Also, evolution of species of the simuliid genus *Gymnopias* [82] and anthomyiid cone seed pests in the genus *Strobilomyia* [83] appears to be associated with the Tertiary Period in Beringia. These examples highlight the applicability of the presented phylogenetic approach to other systems. At the same time, we have to note significant limitations of the molecular clock analysis for the mosquito species — the lack of reliably dated paleontological calibration points and widespread introgression events [7]. These limitations can significantly affect molecular clock estimations based on the single divergence date. Operating at the 95% credibility intervals of the divergence time can be a more reliable approach to the mosquito phylogenomics.

## Conclusions

In this study, we reconstructed the relationships among the Eurasian members of the Maculipennis Group using multigene and chromosome phylogenies. The analysis has demonstrated that genome-wide approaches are highly effective for reconstruction of the evolutionary history of the Holarctic malaria mosquitoes. Our phylogeny provided strong support for the branching order of all Eurasian species and for a basal position of the Southern Eurasian clade with respect to the European and Northern Eurasian clades. We demonstrated that *An. beklemishevi* clusters together with the other Eurasian members and, thus, belongs to the Palearctic Maculipennis Subgroup. Phylogenomic data support migration of the Maculipennis mosquitoes from North America to Eurasia through the Bering Land Bridge ~ 20–25 Mya. Our study demonstrated that the dominant malaria vectors do not form a monophyletic group indicating that vector competence evolved multiple times. Finally, the ability to enter the compete diapause in winter developed independently in different lineages of the Maculipennis Group.

## Methods

### Mosquito species studied, sample collections, and material preservation

The aim of our study was to reconstruct the whole-genome phylogeny of the Maculipennis Subgroup. For this purpose, we included 14 species of malaria mosquitoes: two Nearctic species (*An. freeborni* and *An. quadrimaculatus*) and 12 Palearctic species (*An. artemievi*,* An. atroparvus*, *An. beklemishevi*, *An. daciae*, *An. labranchiae*, *An. maculipennis*,* An. martinius*,* An melanoon*,* An. messeae*, *An. persiensis*, *An. sacharovi*, and *An. sinensis*, an outgroup species). For genome and transcriptome sequencing, we sampled 12 species (*An. artemievi*,* An. beklemishevi*, *An. daciae* (Moscow and Tomsk populations), *An. labranchiae*, *An. maculipennis*,* An. martinius*,* An melanoon*,* An. messeae*, *An. persiensis*, and *An. sacharovi*) and two Nearctic species (*An. freeborni* and *An. quadrimaculatus*) (Additional file 1: Table S3). Thus, whole-genome or transcriptome sequencing was performed for 12 out of 14 species, and the genome sequences of *An. atroparvus* EBRO (AatrE3) and *An. sinensis* (AsinS2) were retrieved from VectorBase [84]. For transcriptome sequencing, blood-free adult females and males of *Anopheles* mosquitoes were fixed in RNAlater to prevent RNA degradation. For genome sequencing, blood-free adult females and males of mosquito species (or larvae of *An. artemievi* and *An melanoon*) were fixed in 95% ethanol for genomic DNA preservation.

### *Anopheles* species identification

Genotyping of ITS2 by Sanger sequencing was performed on the collected mosquitoes to identify species. The body of each sample was homogenized in liquid nitrogen and genomic DNA was extracted using the standard protocol for the Qiagen DNeasy Blood and Tissue Kit (Qiagen, Germantown, MD, USA), followed by DNA elution in 100 µl of water. PCR reactions and primer choice for ITS2 sequencing were conducted in accordance with the previous studies with modifications [45, 85, 86]. Specifically, ITS2 from rDNA was amplified using the forward universal primer annealed to a conserved 5.8S rRNA region (5.8Sseq_for: 5′-ATCACTCGGCTCTCGTGGATCG-3′) [45] and the reverse primer annealed to a conserved 28S rDNA region (ITS2_Anm-d_rev: 5′-ATGCTTAAATTTAGGGGGTA-3′) [85, 86]. PCR mixture contained 1–2 µl (~ 40 ng) of DNA template, 1 µl of 10 mM of each forward and reverse primers, and 10 µl of 2 × ImmomixTM with DNA polymerase (Meridian Life Science, Inc., Memphis, TN, USA). Water was added to the mixture up to 20 µl of the total volume. Amplification was performed using a thermal cycler (Eppendorf, Enfield, CT, USA) with the following programmed parameters: initial denaturation at 95 °C for 10 min, followed by 35 cycles of 95 °C for 15 s, 55 °C for 30 s and 72 °C for 30 s, and a final extension step at 72 °C for 5 min. The reaction was placed on hold at 4 °C. For DNA sequencing, amplicons were checked on a gel and then purified with WizardTM PCR Clean Up Kit (Promega Corporation, Madison, WI, USA). Concentrations of purified PCR products were measured. PCR products were mixed with 3.2 pmol of either forward or reverse primers and Sanger sequenced at the Genomics Sequencing Center of Virginia Tech. The samples were sequenced with both forward and reverse directions to confirm the presence of the same SNPs on both DNA strands. Sequences were trimmed, aligned, and analyzed using a Lazergene software (DNASTAR, Inc., Madison, WI, USA) with modules EditSeq, Seqman Pro, and NCBI BLAST (optimized for highly similar sequences from nucleotide collection). Species identification was done based on the percent identity of the query with the subject sequence of at least 99%. Sequences that match the previously described SNPs, in positions 150, 211, 215, 217, 412, and 432, were used to distinguish the ITS2 sequences of *An. messeae* from *An. daciae* [2].

### Transcriptome and genome sequencing

We performed transcriptome sequencing for *An. messeae*,* An. daciae* (Moscow and Tomsk populations),* An. quadrimaculatus*,* An. beklemishevi*,* An. labranchiae*,* An. maculipennis*,* An. freeborni*, and *An. sacharovi*. Total RNA was isolated from 15 individual specimens from each of the 8 species. The polyA-selection method was used and quality control of the RNA sequencing libraries was performed, including size evaluation by a bioanalyzer and by quantitative assay (HudsonAlpha Institute for Biotechnology Huntsville, AL, USA or Fasteris, Inc., Geneva, Switzerland). Transcriptome sequencing was done using HiSeq 2500 1 × 100 or 1 × 125 bp, depending on the service provider. We received from 8.2 to 12.56 Gb of data for each sample. For the genome sequencing of *An. martinius*,* An. artemievi*,* An. melanoon*, and *An. persiensis*, DNA was extracted from adult or larvae mosquitoes using the DNeasy Blood and Tissue extraction system (Qiagen, Germantown, MD, USA) following the manufacturer’s protocol with slight modifications. Genomic pools were created from 33 individuals of *An. martinius*, 40 individuals of *An. artemievi*, six individuals of *An. melanoon*, and six individuals of *An. persiensis.* The pools were sequenced using the Illumina HiSeq 4000 Platform PE150. We obtained from 50 × to 70 × genome coverage for each species.

### Transcriptome assembly and annotation

The raw reads were trimmed and the remains of adapters were removed using Trimmomatic software [87] with the following settings: *ILLUMINACLIP:2:30:10 LEADING:24 TRAILING:24 SLIDINGWINDOW:4:24 MINLEN:50*. The transcriptomes were assembled using Trinity 2.1.1 software [88] with strand-specific settings. Open reading frames (ORFs) were extracted from the assemblies with Transdecoder utility [89] and translated ORFs were blasted against a collection of mosquito proteins from VectorBase [84] with an *e*-value threshold 1e − 5 using the blastp software [90]. Proteins with blast hits were used in the TransDecoder. Then, the Predict module was employed to find additional coding sequences that did not have any blast hits within datasets based on hidden Markov model (HMM) profiles derived from the training set of proteins homologous to other mosquitoes. To remove redundancy caused by the possible assembly of different isoforms, haplotypes, and fragments of genes, the resulting sets of proteins were clustered using CD-HIT software [91] with a 97% identity threshold *(-c 0.97 -n 5 -M 2000*). Thus, the final sets of proteins consisted of collapsed proteins with either blast hits against the *Anopheles* proteins or predicted using TransDecoder, Predict, or the HMM-based module algorithm. The corresponding mRNA and CDSs were extracted from each species and subsequently used for the downstream analysis. To assess the quality and completeness of the assembled transcriptomes, benchmarking of single-copy orthologues using BUSCO V.1.22 [92] was applied with a Diptera-specific dataset consisting of 2799 single-copy genes and the following settings: *–m OGS –species aedes*.

### Pseudoassembly of whole-genomic sequences

Genomic reads were aligned to the *An. atroparvus* AatrE3 genome using Burrows-Wheeler Aligner (BWA) mem software [93]. The resulting reads were sorted using Samtools [94]. Binary Alignment Map (BAM) files were used to create consensus sequences with the following settings: *samtools mpileup -q 50 -Q 26 | bcftools call | vcfutils.pl vcf2fq -d 8 -D 80*. To reduce possible errors caused by interspecies alignment, stringent criteria were used during the consensus construction including a minimum phred-scaled mapping quality equal to 50 (*-q* 50), a minimal phred-scaled quality of a base to call a substitution equal to 26 (*-Q* 26), and minimum and maximum depths equal to 8 and 80, respectively. The maximum depth threshold was utilized to reduce the likelihood of calling substitutions in highly duplicated or repetitive regions in addition to the mapping quality threshold. During assembly, genomic regions that were not covered or poorly covered were converted into Ns and excluded from subsequent analysis. The consensus transcripts were processed with seqtk utility (https://github.com/lh3/seqtk) to select a random base from heterozygous positions and then translated into amino acid sequences for the orthology inference step.

### Orthology inference and alignment

Amino acid sequences were assigned to the orthogroups using OrthoFinder 1.1.4 software [95] using the total set of 15 species. Orthogroups were manually filtered to exclude orthogroups containing missed species and any paralogs. The nucleotide sequences from the single-copy orthogroups (CDSs) were aligned in the codon-aware mode (*F* + *-codon*) using Prank software [96]. The aligned sequences were concatenated and cleaned using Trimal [97] utility, which removed all codons with any gaps in alignment (*-nogaps*).

### Phylogenetic tree reconstruction

To determine the optimal substitution model for the dataset, jmodeltest2 [98] software and Bayesian Information Criteria (BIC) were utilized. The maximum likelihood tree was constructed using the RAxML 8 package [99] and the GTRGAMMAI model. The topology was tested with 1000 bootstrap replications. The phylogenetic concordance was calculated as a percentage of individual gene trees supporting the species tree topology using the sumtrees.py script of the DendroPY package [100]. The individual gene trees were calculated in a similar way with the species tree but with 200 bootstrap replications. To determine the divergence time of the main Maculipennis lineages, we extracted 126,025 fourfold degenerated sites using MEGA 7 software [101] from the final alignment. The divergence time was estimated using MCMCtree from the PAML 4.8 [102] package using the approximate likelihood calculation and the GTR + G model of nucleotide substitution after 10 million MCMC generations (including the first one million being discarded as a burn-in). We used independent clock rate model, the alpha parameter for gamma rates (*n* = 4) at site was set up at 0.5. Due to the lack of well-established fossil calibrations for the studied and closely related taxonomic groups, we used a root calibration reflected divergence of *An. sinensis* for the rest of the group (from 35 to 45 Mya) based on previous work [6].

### Analysis of introgression

To test our hypothesis for interspecies introgression, we used Hybridcheck software [103]. This software uses well-established statistics: D statistics (ABBA-BABA test) [104] and the f statistic [105] to test hypotheses about phylogenetic discordance caused by putative hybridization events for 4-taxon sets. The significance of the D statistic was tested using the jackknife method with 10 blocks and then corrected using the Bonferroni approach. The software generates all possible 4 taxon trees and calculates statistics for each of them. After the calculation, all significant results were manually checked for correspondence to the maximum-likelihood phylogeny, and discordant trees and redundant species combinations were removed from the analysis.

### Chromosome preparation

Polytene chromosome preparations for fluorescence in situ hybridization (FISH) were prepared from ovarian nurse cells of female malaria mosquitoes from the following species: *An. atroparvus*, *An. beklemishevi*, *An. labranchiae*, *An. maculipennis*, and *An. sacharovi*. Adult mosquito ovaries were dissected and fixed in Carnoy’s solution (3 parts 96% ethanol to 1 part glacial acetic acid). Ovaries were stored at − 20 °C for up to one year before using. For squashed chromosome preparations, 20–30 follicles were put on a glass slide in a drop of ice-cold propionic acid and left for 5 min for maceration. The drop of propionic acid was then changed by a new one, follicles were spread along the slide using needles, covered with an 18 × 18 coverslip, and squashed by tapping the coverslip using the needle handle. Preparations were then covered by a piece of filter paper for additional tapping. The quality of the chromosome spreading was analyzed under phase contrast microscopy using AxioImager A1 (Carl Zeiss, OPTEC, Novosibirsk, Russia) and AxioVision 4.8.1. software (Carl Zeiss, OPTEC, Novosibirsk, Russia). Only high-quality preparations were used for further procedures. Coverslips were removed by a razor blade after freezing the preparation by dipping it in liquid nitrogen and then slides were immediately placed in ice-cold 50% ethanol for 5 min. Subsequently, slides were kept in 70% and 96% ethanol for 5 min and air-dried. After 1-week storage at room temperature, chromosomal preparations were used for FISH.

### *DNA probe preparation and fluorescence *in situ* hybridization*

Exons of 21 selected genes from the *An. atroparvus* genome were used to design primers using the Primer-BLAST tool [106]. Sequences of expected PCR products were matched against the *An. atroparvus* genome using the VectorBase BLAST tool to ensure the uniqueness of the DNA-probes. DNA-probes were amplified by PCR and labeled using the Random Primer Labeling Protocol, as described earlier [107]. The DNA-probes were precipitated in ethanol, dissolved in a hybridization solution (50% formamide, 10% sodium dextran sulfate, 0.1% Tween-20 in 2 × Saline-Sodium Citrate (SSC), pH 7.4), and stored at − 20 until use. FISH was performed following our standard protocol [108, 109]. Microscopic analysis of chromosome preparations after FISH was performed using AxioImager Z1 (Carl Zeiss, OPTEC, Novosibirsk, Russia) equipped with a device for improving images, ApoTome (Carl Zeiss, OPTEC, Novosibirsk, Russia) and a CCD camera MRm (Carl Zeiss, OPTEC, Novosibirsk, Russia). Images were captured and processed using AxioVision 4.8.1. software (Carl Zeiss, OPTEC, Novosibirsk, Russia).

### Multiple Genome Rearrangement analysis

The calculation of inversion distances among the included species of the Maculipennis Group species was performed using the Multiple Genome Rearrangement (MGR) program [52]. The signed option of the MGR program was used. This program implements an algorithm that uses a parsimony approach, *i.e*. it minimizes the sum of the rearrangements over all the edges of the phylogenetic tree [52]. To create an inversion phylogenetic tree, numbers were assigned to represent each conserved synteny block in species using our gene mapping data.

## Supplementary Information

**Additional file 1: Table S1. **Statistics of the transcriptome assemblies for species of the Maculipennis Group. **Table S2. **Genes of *An. atroparvus* used as markers for detection of interspecies chromosome rearrangements on the X chromosome in five species of the Maculipennis Subgroup. **Table S3. **Mosquito species and sampling sites. **Figure S1. **A phylogenetic analysis of ortholog groups split into 4 equally-sized datasets based on the length of the alignments after *trimal* filtration. **Figure S2.** A phylogenetic analysis using whole-genome datasets from six *Anopheles* species.

## Data Availability

The data and materials are available from the GenBank repository under the BioProject accession number PRJNA861430 [110].

## Abbreviations

BWA: Burrows-Wheeler Aligner
CDS: Coding sequence
FISH: Fluorescence in situ hybridization
HMM: Hidden Markov model
Mya: Million years ago
MGR: Multiple Genome Rearrangements
SNP: Single nucleotide polymorphism
