# Supplementary material for: Phylogenomics revealed migration routes and adaptive radiation timing of Holarctic malaria mosquito species of the Maculipennis Group
Source: BMC Biol. 2023 Apr 10;21:63. doi: 10.1186/s12915-023-01538-w (PMC10084679; doi:10.1186/s12915-023-01538-w)
Supplement: Supplementary file 1 — Additional file 1: Table S1. Statistics of the transcriptome assemblies for species of the Maculipennis Group. Table S2. Genes of An. atroparvus used as markers for detection of interspecies chromosome rearrangements on the X chromosome in five species of the Maculipennis Subgroup. Table S3. Mosquito species and sampling sites. Figure S1. A phylogenetic analysis of ortholog groups split into 4 equally-sized datasets based on the length of the alignments after trimal filtration. Figure S2. A phylogenetic analysis using whole-genome datasets from six Anopheles species. [file 12915_2023_1538_MOESM1_ESM.pdf]

Additional file 1.

Table S1. Statistics of the transcriptome assemblies for species of the Maculipennis Group.

Species	Total proteins	CDS N50, bp	Average CDS, bp	Total assembled bases	BUSCO
An. beklemishevi	15135	681	620.04	9384156	C:28.2%[S:28.0%,D:0.2%],F:21.8%,M:50.0%
An. daciae Moscow	21658	1014	803.73	17407329	C:56.2%[S:54.3%,D:1.9%],F:18.6%,M:25.2%
An. daciae Tomsk	21544	1212	907.56	19552251	C:68.2%[S:66.1%,D:2.1%],F:14.7%,M:17.1%
An. freeborni	19832	1299	986.58	19565613	C:69.4%[S:67.4%,D:2.0%],F:17.4%,M:13.2%
An. labranchiae	14404	813	691.62	9962130	C:36.7%[S:36.5%,D:0.2%],F:21.0%,M:42.3%
An. maculipennis	17003	930	757.8	12884655	C:47.4%[S:47.1%,D:0.3%],F:21.4%,M:31.2%
An. messeae	22236	1086	835.62	18580944	C:62.1%[S:60.0%,D:2.1%],F:17.1%,M:20.8%
An. quadrimaculatus	22052	1329	974.73	21495072	C:73.3%[S:69.7%,D:3.6%],F:12.8%,M:13.9%
An. sacharovi	16817	1290	957.81	16107663	C:64.1%[S:63.8%,D:0.3%],F:17.2%,M:18.7%

Table S2. Genes of *An. atroparvus* used as markers for detection of interspecies chromosome rearrangements on the X chromosome in five species of the Maculipennis Subgroup.

	Gene ID	Gene start (bp)	Gene end (bp)	Distance from the previous gene (bp)*	Supercontig	Chromosome band
1	AATE010696	18023	22191		KI421896	1A
2	AATE001169	1162806	1170169	1140615	KI421896	1A
3	AATE017741	1960425	1963110	790256	KI421896	1B
4	AATE010870	2927563	2928974	964453	KI421896	1C
5	AATE017428	3964271	3982124	1035297	KI421896	2A
6	AATE012020	4602182	4608814	620058	KI421895	2A
7	AATE015765	5527302	5531343	918488	KI421895	2B
8	AATE000795	6504256	6513151	972913	KI421895	2B
9	AATE002125	7208125	7217523	694974	KI421895	2C
10	AATE004111	8538061	8542551	1320538	KI421895	2C
11	AATE001403	8751285	8754177	208734	KI421895	2C
12	AATE018270	9855878	9857040	1101701	KI421907	3B
13	AATE017493	10389518	10398371	532478	KI421907	3B
14	AATE021170	11446721	11457749	1048350	KI421907	3C
15	AATE018218	11958281	11959687	500532	KI421898	3C

16	AATE005236	12437092	12444184	477405	KI421898	3C
17	AATE010434	13484949	13487325	1040765	KI421898	4A
18	AATE009010	14525064	14530105	1037739	KI421898	4A
19	AATE018661	15491805	15495758	961700	KI421898	4A
20	AATE010210	15918625	15921507	422867	KI421919	4B
21	AATE016157	16324241	16326290	402734	KI421920	4B

*The distance between genes was calculated as the number of nucleotides from the start to the end of a gene on the *An. atroparvus* genome map.

Table S3. Mosquito species and sampling sites.

**Species**	**Life stage**	**Country**	**Region**	**Location**	**GPS coordinates**	**Date of collection**
An. artemievi	larvae	Kyrgyzstan	Osh	Kydyrsha and Kyzyl Shark	40.726613, 72.957595; 40.725281, 72.932879	08/22/2006
An. beklemishevi	adults	Russia	Tomsk	Chainsk	57.931778, 82.596862	08/22/2016
An. daciae Moscow	adults	Russia	Moscow	Novokosino	55.734170, 37.843453	08/28/2015
An. daciae Tomsk	adults	Russia	Tomsk	Kandinka	56.292605, 84.806183	07/08/2015
An. freeborni	adults	USA	California	Marysville	Unknown (colony)	Established in 1943
An. quadrimaculatus	adults	USA	Florida	Orlando	Unknown (colony)	Established in 1939
An. labranchiae	adults	Italy	Tuscany	Princhipina terra	42.724717, 11.041467	08/02/2015
An. maculipennis	adults	Italy	Lacio	Rieti	42.404833, 12.829150	08/05/2015
An. martinius	adults	Kazakhstan	Kysyl-Orda	Kyzil-Orda	44.852417, 65.118217	08/23/2005
An. melanoon	larvae	Georgia, Abkhazia	Gudauta	Primorskiy	43.09351, 40.69351	07/04/2017
An. messeae	adults	Russia	Moscow	Novokosino	55.734170, 37.843453	08/28/2015
An. persiensis	adults	Iran	Mazandaran	Alendan, Andar-Gholi, Chader-Mahaleh, Chalmardi	36.221983, 53.433833; 36.339467, 52.883750; 36.513650, 52.345100; 36.561389, 53.392500	09/07/2017 09/06/2017 05/19/2017
An. sacharovi	adults	Armenia	Ararat	Araksavan	39.784329, 45.229700	09/02/2016

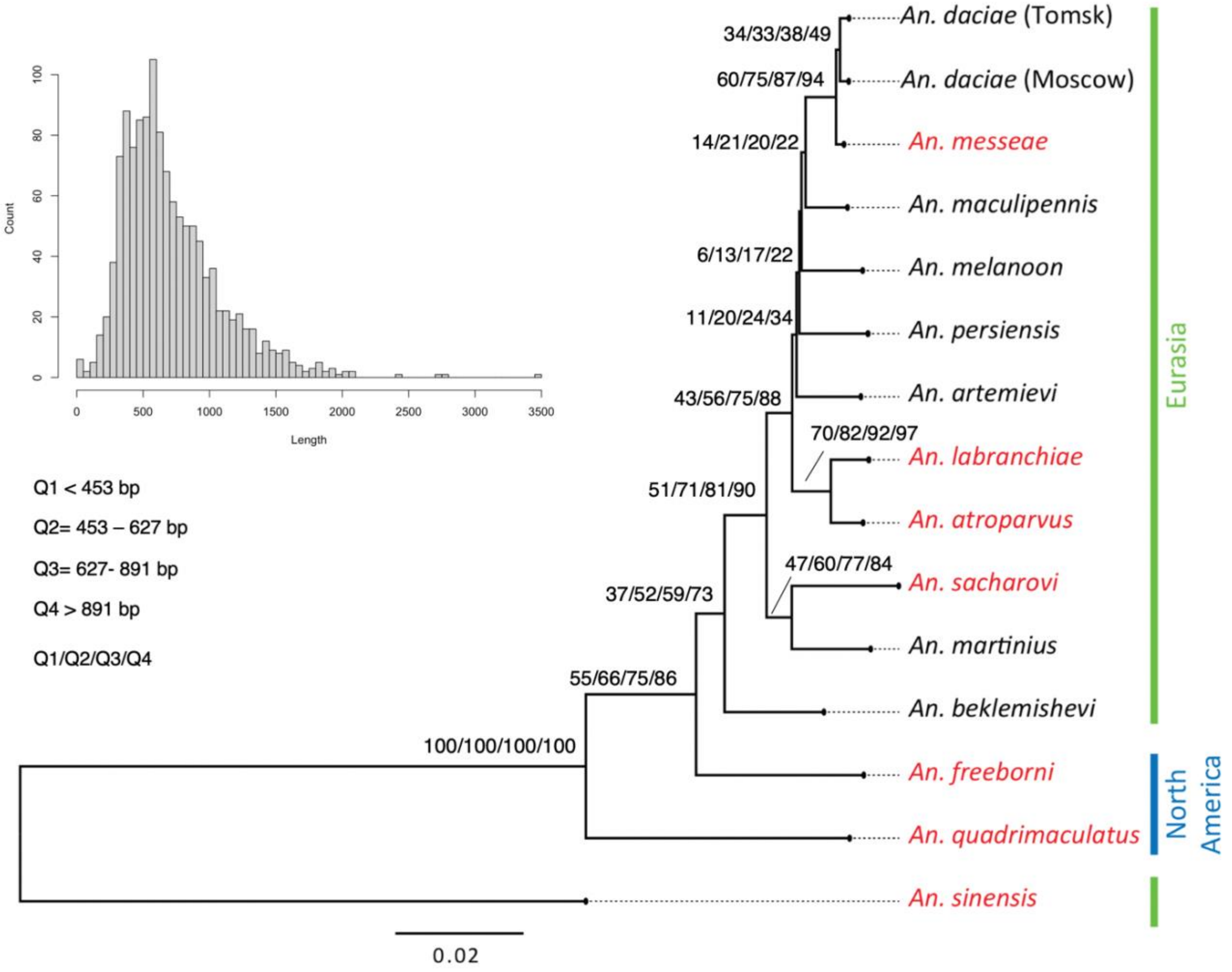

Figure S1. A phylogenetic analysis of ortholog groups split into 4 equally-sized datasets based on the length of the alignments after *trimal* filtration. The values indicate the fraction of the trees which support given split for the groups Q1, Q2, Q3 and Q4 with the increasing length of the alignment. The long alignments contain more polymorphic and parsimony informative sites and better resolve phylogenetic incongruences. At the same time, the nodes with the lowest concordance are attributed to the group of species with high evidence of introgression events (*An. persiensis*, *An. melanoon*, *An. maculipennis*, *An. messeae*). This approach is based on the single copy orthologs and very diverse set of species with long evolutionary history. Both introgression events and unequal phylogenetic signal among the taxons for genes of different length could influence the observed concordance values. Future studies with high-quality assembled genomes of the studied species will allow to better understand the complex interplay of phylogeny, introgression, recombination and fading phylogenetic signal with decreasing divergence time.

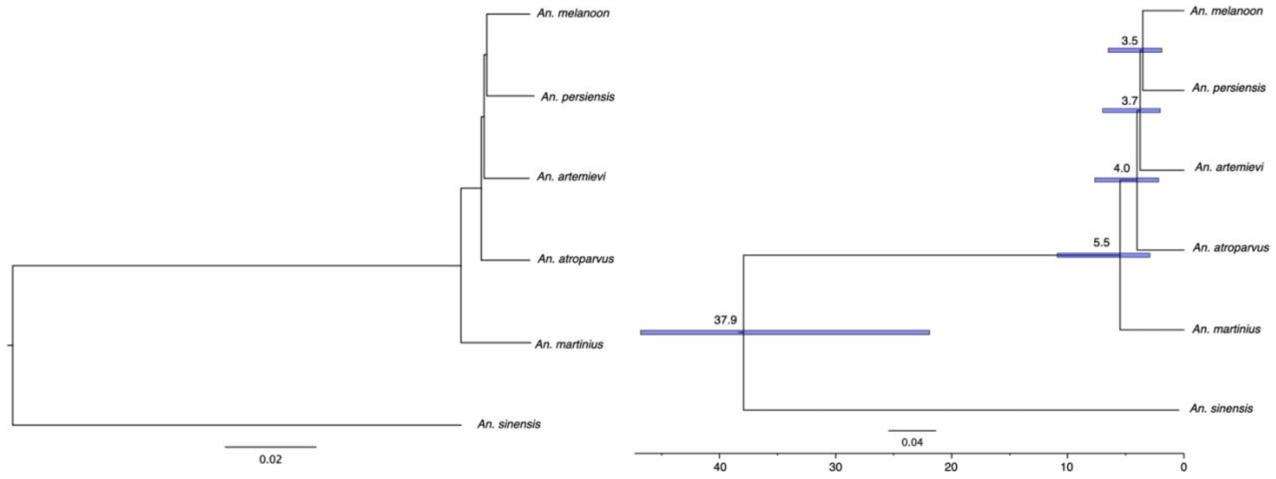

Figure S2. A phylogenetic analysis using whole-genome datasets from six *Anopheles* species. The topology of the tree is identical to the trees in Figures 2 and 3 of the manuscript. The estimation of the divergence time for insects is a problematic task because of the lack of the reliable paleontological calibration points. This analysis used previously established divergence time of the *An. sinensis* split from the rest of the species (35-45 Mya). The root divergence 95% interval becomes much wider with the 6-taxon scheme than in the manuscript and the divergence time of the main group becomes younger in general although with wider 95% intervals. A scale bar refers to a phylogenetic distance in a fraction of nucleotide differences. The time scale is in Mya, mean values and time intervals are indicated in blue above the branches.
